# Effectiveness of focused structural massage and relaxation massage for chronic low back pain: protocol for a randomized controlled trial

**DOI:** 10.1186/1745-6215-10-96

**Published:** 2009-10-20

**Authors:** Daniel C Cherkin, Karen J Sherman, Janet Kahn, Janet H Erro, Richard A Deyo, Sebastien J Haneuse, Andrea J Cook

**Affiliations:** 1 Group Health Research Institute, Seattle, USA; 2 College of Medicine, University of Vermont, Burlington, USA; 3 School of Medicine, Oregon Health and Science University, Portland, USA

## Abstract

**Background:**

Chronic back pain is a major public health problem and the primary reason patients seek massage treatment. Despite the growing use of massage for chronic low back pain, there have been few studies of its effectiveness. This trial will be the first evaluation of the effectiveness of relaxation massage for chronic back pain and the first large trial of a focused structural form of massage for this condition.

**Methods and Design:**

A total of 399 participants (133 in each of three arms) between the ages of 20 and 65 years of age who have low back pain lasting at least 3 months will be recruited from an integrated health care delivery system. They will be randomized to one of two types of massage ("focused structural massage" or "relaxation massage"), or continued usual medical care. Ten massage treatments will be provided over 10 weeks. The primary outcomes, standard measures of dysfunction and bothersomeness of low back pain, will be assessed at baseline and after 10, 26, and 52 weeks by telephone interviewers masked to treatment assignment. General health status, satisfaction with back care, days of back-related disability, perceived stress, and use and costs of healthcare services for back pain will also be measured. Outcomes across assigned treatment groups will be compared using generalized estimating equations, accounting for participant correlation and adjusted for baseline value, age, and sex. For both primary outcome measures, this trial will have at least 85% power to detect the presence of a minimal clinically significant difference among the three treatment groups and 91% power for pairwise comparisons. Secondary analyses will compare the proportions of participants in each group that improve by a clinically meaningful amount.

**Conclusion:**

Results of this trial will help clarify the value of two types of massage therapy for chronic low back pain.

**Trial registration:**

Clinical Trials.gov NCT 00371384.

## Background

Massage has become one of the most popular complementary and alternative medical (CAM) therapies for back pain, the condition for which CAM therapies are most commonly used [1]. Furthermore, back and neck pain are the most common reasons for which massage care is sought, representing more than one third [2] of the more than 100 million annual visits to massage therapists [3]. As currently practiced in the U.S., massage incorporates a variety of soft-tissue techniques and sometimes includes techniques aimed at changing how patients perceive and use their bodies in daily activities. Virtually all massage schools teach basic Swedish massage techniques aimed at relaxation, but few offer substantial training in techniques for treating chronic musculoskeletal pain as part of the basic training program. Only a minority of massage therapists take continuing education courses in these techniques.

Prior to 2000, there were no published trials evaluating the effectiveness of any types of massage for back pain. By the time the current study was proposed (October, 2004), one medium-sized randomized clinical trial (RCT) (78 massage subjects) [4] and two small RCTs (12 and 25 massage subjects) [5,6] found back-pain focused massage effective for subacute and chronic back pain at the end of treatment. The only trial that measured long-term outcomes found that the benefits of massage were still evident 9-10 months after completion of therapy [4]. In contrast, there have been no published evaluations of the effectiveness of relaxation massage for back pain, despite its being the most widely available type of massage in the United States.

If relaxation massage were found to be an effective treatment for chronic back pain, it might provide a valuable non-pharmacologic alternative to commonly prescribed muscle relaxants, which are of questionable benefit for chronic back pain and which have significant side effects and risk of habituation [7,8]. However, if relaxation massage is not effective, patients with chronic back pain wishing to try massage should be directed to the minority of massage therapists adequately trained in therapeutic massage techniques, and massage schools would do well to include such training in their basic programs.

The primary aim of this study is to determine if Relaxation Massage, employing Swedish massage techniques, is an effective treatment for chronic low back pain in terms of reduced pain and improved function. *We hypothesize that Relaxation Massage will be more effective than continued usual care alone*.

Our secondary aims are:

a) To determine if Focused Structural Massage, employing neuromuscular and myofascial techniques, is more effective than Relaxation Massage. *We hypothesize that Focused Structural Massage will be more effective than Relaxation Massage*.

b) To explore the ability of pre-treatment psychosocial and clinical characteristics to predict which patients will benefit most from each type of massage.

## Methods and Design

### Overview

**Figure 1:**
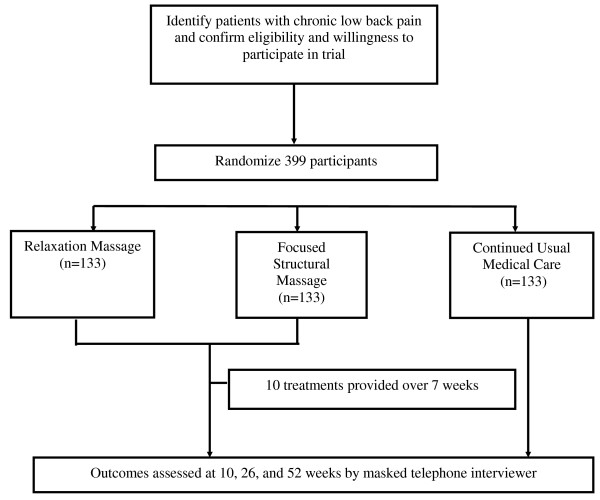
**Study Design**. Recruitment, randomization to treatment, and outcomes assessment.

We will recruit health plan patients who have sought medical care for chronic low back pain. Eligible participants will be randomized equally to one of three groups (Figure 1): Relaxation Massage (RM), Focused Structural Massage (FSM) or continued usual care (UC). Participants in the two massage arms will receive 10 treatments over a 10-week period. The massage treatments will be provided by licensed massage therapists with experience in both RM and FSM and who will receive special training in implementation of the treatment protocols. To minimize disappointment (and possibly losses to follow-up), participants assigned to UC will receive $50. All participants will retain access to the health care services to which they are entitled by their insurance coverage.

Participants will be followed for a one-year period following randomization and primary and secondary outcomes will be assessed by telephone interviewers masked to treatment assignment after 10, 26, and 52 weeks. The primary outcomes will be dysfunction due to back pain and bothersomeness of back pain. Secondary outcomes will include general health status, satisfaction with back care, days of restricted activity, perceived stress, and use and costs of back-related health care for the year following randomization.

Bias will be minimized by a clinical protocol designed to offer maximum possible masking in a study of a physical procedure such as massage. Participants in the two massage groups will know only that they will receive one of two methods of massage for treating back pain and that it isn't known which method is more effective. Furthermore, outcomes data will be collected by telephone interviewers masked to treatment assignment.

We will perform an intention-to-treat analysis of the data, i.e., the analysis will be by randomized assignment regardless of participation in treatment sessions. We will then analyze psychosocial data collected at baseline to explore whether any of the baseline characteristics predict clinically significant improvement from: 1) massage versus no treatment and 2) from one type of massage treatment but not the other. In addition, we will determine which specific FSM treatments emphasized by massage therapists' (as recorded on the visit treatment forms) are associated with the best treatment outcomes.

### Study population

This study will focus on patients between 20 and 65 years of age with non-radicular chronic low back pain of mechanical origin (as opposed to infectious, neoplastic, or inflammatory causes). There are many causes of low back pain, but in most cases, the precise pathoanatomic diagnosis is unattainable because of the weak association among symptoms, pathoanatomic changes, and imaging results.

### Inclusion and exclusion criteria

Health plan members between 20 and 65 years of age with ICD-9 diagnoses indicative of non-specific low back pain will be eligible for the study if their pain has lasted at least three months, they rate their low back pain at least 3 on a 0 to 10 back pain bothersomeness scale, and give informed consent (Tables 1 and 2). Reasons for exclusion will be identified from two sources: 1) automated data on ICD-9 diagnoses recorded during all visits over the previous year made by health plan members identified with low back pain-compatible ICD-9 diagnoses, and 2) telephone eligibility interviews. Members found to have any of the following will be excluded: structural or neurologic causes or potential causes of low back pain (i.e., sciatica, underlying systemic or visceral disease, pregnancy, spondylolisthesis, spinal stenosis, cancer or unexplained weight loss, recent vertebral fracture); inappropriate candidate for massage (e.g., the chronic conditions identified in Tables 1 and 2); characteristics complicating the interpretation of findings (i.e., involved with litigation or compensation claim for back pain, evidence of severe or progressive neurologic deficits, back surgery within the prior three years, planning to seek other treatment for back pain); or characteristics related to ability to complete the study protocol (i.e., unable to speak or read English, plans to move out of town).

**Table 1:** Inclusion Criteria

**Inclusion criteria**	**Rationale**	Source*
Continuing member of Group Health	Defined population that is easy to identify, recruit and follow-up and who have been evaluated by a Group Health physician	A, TI

20 through 64 yrs of age	Chronic low back pain in children results from different causes than those we are studying; Older people have higher risk of undiagnosed serious conditions causing low back pain	A

At least one primary care visit for back pain within the past 3-12 months	Efficient method for identifying people who may have chronic low back pain	A

Non-specific, uncomplicated low back pain, i.e., these ICD-9 codes:	These codes are consistent with low back pain that is uncomplicated and mechanical in nature	A
		
724.2 Lumbago 724.5 Backache, unspecified 724.8 Other symptoms referable to back 846.0-9 Sprains and strains, sacroiliac 847.2 Sprains and strains, lumbar 847.3 Sprains and strains, sacral 847.9 Sprains and strains, unspecified site of the back		

Physician willing to have patients included in the study	Research policy	**

Lives or attends primary care clinic within 45 minutes travel time from a study massage therapist	Maximize compliance with treatment protocol requiring 10 visits	A

*A = Automated visit data; TI = Telephone interview

** = determined by letter to physician before the sample identification

**Table 2:** Exclusion Criteria

**Exclusion criteria**	**Rationale**	Source*
Cancer (other than basal cell or squamous cell cancer of skin)	Back pain due to, or possibly result of, specific disease/condition	A, TI
Discitis		A
Disk disease		A
Fracture of vertebra		A, TI
Infectious cause of back pain		TI
Scoliosis, severe or progressive		A, TI
Spinal stenosis		A, TI
Osteoporosis		A, TI
Spondylolisthesis		TI

Sciatica	Back problem of complicated nature, including medico-legal issues	TI
Seeking/receiving compensation/litigation for back pain		TI
Surgery, recent, in the past 6 months		A, TI
Surgery, previous low back, within 3 years		TI

Pregnancy	Safety not confirmed	TI

Stroke, recent in the past 6 months	Condition might make it difficult to receive or complete the treatments	A
Paralysis		A, TI
Physically unable to undergo massage sessions (e.g., cannot lie prone for 45 minutes)		TI
Psychoses, major		A, TI
Schedules do not permit attending treatment sessions at times they are offered		TI
Vision problems, severe		TI
Hearing problems, severe		TI
Lack of transportation		TI

Seizure disorder	Condition might contribute to increased risk of severe adverse event	A, TI

Fibromyalgia, severe	Condition/circumstance might confound treatment effects or interpretation of data	TI
Rheumatoid arthritis/Ankylosing spondylitis		A, TI
Other disabling chronic conditions (e.g., disabling heart or lung disease, diabetic neuropathy, receiving treatment for hepatitis)		TI
Planning on seeing health care provider other than primary care provider for low back pain		TI

Dementia	Condition would make it difficult for fully informed consent	A
Unable to read or speak English		TI

Has had massage in the past 12 months for any reason	Wants massage for free, or potential for preconceived notions about massage possibly confounding data	TI

Low back pain has lasted < 3 months	Low back pain not chronic	TI

Bothersomeness of pain score < 3	Back pain too mild to be able to detect improvement	TI

Hypersensitivity to touch or loss or sensation	Inappropriate conditions for massage	TI
Cardiovascular compromise		A, TI
Deep vein thrombosis		A, TI

*A = Automated visit data

*TI = Telephone interview

Because sciatica is subjective we will ask patients about radiating pain, any association with numbness or tingling, whether it radiates below the knee, whether it is worse with coughing or sneezing, and whether the leg pain is worse than the back pain. These responses are combined to make a decision whether or not sciatica is likely. To minimize the likelihood of including persons who have strongly preconceived notions about specific massage treatments or who wish to participate in this trial only to obtain free massage treatments that they were planning to pay for anyway, we will exclude potential participants who have received massage for any reason within the past 12 months. Finally, we will require that participants be willing to visit one of the study massage therapists during the 10-week treatment period and to respond to the 3 follow-up questionnaires.

### Recruitment procedures

Group Health members with in-plan visits resulting in diagnoses of non-specific low back pain will be identified from automated visit data. Those with diagnosis codes during the prior year that correspond to the exclusion criteria will be excluded. Primary care physicians will be provided with a description of the study and given an opportunity to ask that their patients not be invited to participate. Three months after their back pain visits, potential participants will be mailed brochures describing the study and key eligibility requirements and inviting participation. The brochure will contain a tear-out pre-paid postcard on which persons interested in the study can write their names and contact information and mail back to our study staff. An interviewer will then phone the members to answer questions and determine eligibility using a computer program to guide the members through a series of screening questions. The screening process ends with documentation in a database of either eligibility or ineligibility. If eligible, the study staff guides the patient through the consent and HIPAA processes and then mails prospective participants who remain interested two signed copies of the combined consent/HIPAA form. The prospective participant will then be asked to sign and return one copy of each form to the study staff in a pre-paid return envelope. Upon receipt of the signed form, a Research Specialist (RS) will contact the potential participant to administer the baseline questionnaire using a computer-assisted telephone interviewing program. Those still willing to participate will be randomized to one of the three treatment arms.

If necessary, we will supplement recruitment by recruiting members who have had recurrent visits for low back pain at any time over the past few years or by placing advertisements in the health plan's quarterly magazine.

### Randomization to treatment groups

After administering the baseline questionnaire, the RS will use an ACCESS database to randomize one third of the participants to usual care and two-thirds to a massage intervention. The RS will ask the participants randomized to a massage treatment about their preference for geographic location of a therapist. The RS will then identify a therapist in the chosen location and enter that therapist's study identification code into the ACCESS database program that will use a pre-programmed computer-generated sequence of blocked random numbers for each therapist to assign the participant, with equal probability, to FSM or RM treatment. Thus, using this two-stage randomization procedure, roughly one-third of all participants will be randomized to each of the three arms. This will also ensure that each massage therapist sees approximately equal numbers of participants in each of the two massage groups. The one-third of participants randomized to the control group will be mailed checks for $50. The randomization database will be built to ensure that treatment allocation cannot be viewed prior to randomization and cannot be changed after randomization.

Participants randomized to the control group will be thanked for their willingness to participate, reminded of the importance of completing the follow-up interviews, and told they will be mailed checks for $50 in 4 to 6 weeks. Shortly after randomization to massage, the RS will call the massage therapist to provide the participant's contact information and to specify which type of massage the participant is to receive. The RS will also give the massage therapist a date by which the first appointment needs to be scheduled for that participant. Participants randomized to a massage treatment will be telephoned within 48 hours by the chosen massage therapist to set up the initial appointment. The massage therapist will call the research assistant to confirm that the first appointment has been made. The massage therapist will provide participants directions to the therapist's office. The massage therapist will schedule the nine subsequent visits with the participant.

### Study Treatments

Participants will be randomized to one of three groups: RM, FSM or UC. The RM and FSM treatment protocols will maximize the distinction between the types of relaxation massage and focused structural massage employed by licensed practitioners but preserve the massage therapists' belief that they are providing potentially helpful treatment by allowing them enough latitude to tailor their treatments to each patient. The massage therapists will carefully document the treatments they actually provide using specially-designed treatment visit forms color-coded and customized for each type of massage. The therapists will provide treatments to participants in both the groups in their offices. Both massage treatment protocols will prescribe 10 visits over 10 weeks, with each follow-up visit lasting one hour. The first visit will be 75 to 90 minutes to allow for a full assessment as well as a treatment lasting 50 to 60 minutes.

In addition to providing the massage, therapists may prescribe self-care exercises from a pre-defined list of seven suggestions, which are designed to enhance the effects of the hands-on treatment. Six of these suggestions are common to both types of massage (see below). Massage therapists will demonstrate each self-care suggestion they make and ask the participant to practice the suggestion. In addition, participants will be provided a standardized handout describing and illustrating each suggestion.

#### Relaxation Massage

In this arm, the massage therapist will give a series of 10 full body massages intended to ease low back pain and improve function by inducing a generalized sense of relaxation.

a) Six distinct techniques will be permitted:

i) Effleurage (gliding)

ii) Petrissage (kneading)

iii) Friction (circular only)

iv) Vibration

v) Rocking and jostling

vi) Holding

b) Time Parameters for Body Areas - Since this is designed as a full-body massage, therapists are required to stay within the following time parameters for particular sections of the body:

i) Head/neck: 5-15 minutes

ii) Back and buttocks: 7-20 minutes

iii) Back of legs: 3-8 minutes

iv) Feet: 2-8 minutes

v) Arms: 5-8 minutes

vi) Hands: 3-8 minutes

vii) Front and sides of legs: 2-10 minutes

viii) Chest and abdomen: 5-15 minutes

c) Self-care suggestions: Following each treatment session, therapists may choose up to 3 suggestions for participants from the following list:

i) Resting Position to Ease the Lower Back

ii) Stretch for Before Getting Out of Bed

iii) Cat-Cow Stretch

iv) Lateral Bend

v) Walking to Help the Lower Back

vi) Golf Ball Roll to Stretch the Plantar Fascia

vii) Conscious Relaxation exercises on a compact disc (unique to the RM treatment protocol)

These self-care suggestions could vary from week to week.

#### Focused Structural Massage

In this arm, the massage therapist will give a series of 10 massages intended to identify and alleviate musculoskeletal contributors to participants' low back pain.

a) Techniques Allowed: A wide range of therapeutic massage techniques will be allowed at the therapists' discretion. However, Swedish massage techniques, which are in prominent use in the RM protocol, can only be used to warm/prepare the tissue or to serve as transition strokes. Specific techniques that are permitted include the following:

i) Postural and palpatory assessment of tissues before, during and after massage.

i) Swedish massage - ONLY to warm and prepare the tissues or to serve as transition strokes

ii) Cross fiber and longitudinal friction massage - believed to break-up adhesions, address fibrosis, and/or increase local circulation in ischemic tissues.

iii) Myofascial Techniques including

(1) Muscle stripping - continuous strokes within the muscle bellies with the goal of separating adherent fascicles, improving circulation, identifying contraction modules, and elongating tissues.

(2) Myofascial release techniques: including direct and indirect, active and passive applications, skin rolling techniques, broad, horizontal and longitudinal plane techniques, pin and stretch (fascia and/or muscle specific) and fascial unwinding.

iv) Neuromuscular Techniques

(1) Sustained compression (ischemic compression) - used to work on active and latent trigger points or tender tissues. Tissues may be placed in neutral, stretched, or slackened position to access the irritable area.

(2) Trigger point therapy

(3) MET (Muscle energy techniques):

(a) post-isometric relaxation

(b) concentric and eccentric contraction based resistives

(c) reciprocal inhibition

(4) Origin insertion approximation technique and stretch applied at muscle attachments

(5) PRT (positional release techniques):

(a) functional technique

(b) facilitated positional release

(c) strain counterstrain

(d) orthobionomy

(6) PNF (Proprioceptive Neuromuscular Facilitation) and proprioceptive stimulation

(7) Lymphatic techniques for relief of swelling, congestion and edema found in involved tissues

(8) Craniosacral Techniques that are part of a biomechanical approach - working with the bones, ligaments, muscles, and fascia of the craniosacral system. Energetic Craniosacral techniques are not allowed.

b) Areas of the body that can be treated - The FSM is expected to vary from one client to another and one session to another, as it is to be used in response to tissue abnormalities, postural habits, and other musculoskeletal impairments that may be contributing to the participant's low back pain. Any area of the body may be treated so long as it is within the scope of practice for Massage Therapists in the State of Washington. It is not assumed, however, that this will necessarily result in full-body massages.

c) Self-care suggestions: Therapists may recommend up to 3 exercises for participants following each treatment session from the following list:

i) Resting Position to Ease the Lower Back

ii) Stretch for Before Getting Out of Bed

iii) Cat-Cow Stretch

iv) Lateral Bend

v) Walking to Help the Lower Back

vi) Golf Ball Roll to Stretch the Plantar Fascia

vii) Psoas Stretch (unique to the FSM treatment protocol)

### Massage therapists

All massage treatments will be performed by licensed massage therapists who have at least 5 years experience, are comfortable strictly following both treatment protocols, and are trained and experienced in the techniques permitted in both the RM and FSM treatment protocols. Approximately 25 massage therapists in the Puget Sound region of western Washington will be recruited from within the network of alternative providers contracted to provide services to Group Health members. This will ensure that the therapists have already met minimum qualifications (e.g., massage training and licensure, malpractice insurance coverage, accessible and adequate treatment facilities). Participating massage therapists will need to agree to strictly adhere to the treatment protocols, be comfortable providing either massage treatment to any of the study participants, and to complete the training program for the study.

### Training and monitoring of massage therapists

The massage therapists will attend 1.5 day training program with co-investigators Sherman and Kahn, and consultant Diana Thompson, an experienced massage therapist who is a national leader in developing standards of care for the profession. This training will include a thorough discussion of the protocol and instruction in using terms consistently when they record the findings of their assessments and the treatments they provide on the treatment visit forms. There will also be hands-on training sessions to ensure that study massage therapists are comfortable with following both of the protocols and with the types of questions they might be asked by participants about the protocols. The therapists will also practice completing the treatment forms correctly and giving self-care suggestions. The training sessions will include "dress rehearsals" for both of the treatment styles using mock participants.

### Assessment of outcomes

A core set of recommended outcome measures [9] covering five important domains will be assessed: back-related dysfunction, pain, general health status, disability and patient satisfaction. The primary effectiveness outcomes are pain and dysfunction assessed at the 10-week telephone interview. Relative effectiveness will be determined by comparing changes from baseline to follow-up among the three treatments arms. Table 3 summarizes the categories of questions included in the baseline and follow-up questionnaires.

**Table 3:** Content of baseline and follow-up questionnaires

**Measures**	**Baseline**	**10-Week**	**26-Week**	**52-Week**
Sociodemographic characteristics	x			

Body mass index (height, weight)	x			

Current smoking status	x			

Back pain history	x			

* Roland Disability Questionnaire (dysfunction)	x	x	x	x

* Bothersomeness of low back pain	x	x	x	x

Satisfaction with back care	x	x	x	x

General Health Status (SF-12)	x	x		x

Disability days	x	x	x	x

Medication use	x	x	x	x

Worry about back problem	x	x	x	x

Exercise (Back-related, general)	x	x	x	x

Confidence in ability to self-manage future back pain	x	x		x

Perceived Stress (PSS)	x	x	x	x

Fear Avoidance (Tampa Scale)	x	x	x	x

Psychological Distress (MHI-5)	x	x	x	x

Global Rating of Improvement		x	x	x

Expectations of treatment	x			

Knowledge of massage	x			

Adverse experiences		x		

Perceptions of massage experience (massage arms only)		x	x	x

Use of non-health plan services for back pain		x	x	x

Use and cost of health plan services for back pain [from automated databases]				

* Co-primary outcome measures

### Primary measures of effectiveness

The modified Roland-Morris Disability Questionnaire will be used to measure back-related patient dysfunction [9,10]. This instrument, which asks 23 yes/no questions and takes approximately five minutes to complete, is scored by summing up the number of "yes" responses. It has been found to be reliable, valid and sensitive to clinical changes [10-12] and is well suited for telephone administration. This is the single most important outcome of the study.

Because there are individuals who are very bothered by even a small amount of pain and others who are not bothered by even moderate pain, our primary measure of symptoms assesses participants' perceptions of the impact of pain on their lives rather merely assigning a pain severity score. Thus, participants will be asked to rate how bothersome their low back pain has been during the past week on a 0 to 10 scale where 0 represents "not at all bothersome" and 10 "extremely bothersome". This measure appears to have substantial construct validity - i.e., it is highly correlated with measures of function and other outcome measures [10].

Both primary outcomes will be measured at baseline and during the 10, 26 and 52 week follow-up interviews. The trial's primary endpoint will be the 10 week follow-up, immediately after completion of the 10 massage treatments. All interviews will be conducted using computer-assisted telephone interviews (CATI)

### Secondary outcome measures

*General health status *will be measured using the well-validated SF- 12 [13] that has been recommended for use in studies of back pain [14]. We will calculate the SF-12 physical and mental health summary scores.

*Days of restricted activity due to back problems*, a surrogate for disability, will be measured with a back-pain specific modification of 3 National Health Interview Survey questions about the number of half-days spent in bed, home from work or school, or cutting down on usual activities due to illness or injury during the past week [15].

Patient global rating of improvement will be measured by asking participants to provide a global rating of improvement in their back-related dysfunction on a seven point scale ranging from "completely gone" to "much worse". Satisfaction with information given about the cause of the back problem, treatments received, and overall care will be measured using three separate questions, each using a 5-point Likert scale (ranging from very satisfied to very dissatisfied).

We will include the following psychosocial measures:

*Psychological distress *will be measured with the 5-item Mental Health Index of the SF-36 [16]. This scale, which assesses general mental health, including depression, anxiety, behavioral-emotional control, and general positive affect, is brief and reliable and has shown good agreement with more comprehensive measures of mental health [17].

*Perceived stress *will be measured with the 10-item version of the Perceived Stress Scale [18], the most widely used self-report measure of psychological stress. The Perceived Stress Scale is a state measure that usually asks about stress experienced over the last month, and is influenced by factors that vary such as daily hassles, life events, appraisals and coping resources [19]. Thus, the PSS should be sensitive to changes in life stress due to the massage intervention.

*Fear avoidance *will be measured with the Tampa Scale for Kinesiophobia [20] that measures back pain patients' fears of movement, exercise and serious underlying disease. We will use the 10-item version that retains acceptable internal consistency (alpha = 0.76), is easier and quicker to administer and has proved sensitive in detecting intervention effects in clinical trials [21]. Typical items (rated on a "strongly agree" to "strongly disagree" Likert scale) are: "I'm afraid that I might injure myself if I exercise;" and "My body is telling me I have something dangerously wrong."

### Measures of health care resource use

Participants' use of health care for back pain during the year following randomization will be measured using automated utilization data, interview data on out-of-plan utilization, and records for each visit to the study massage therapists. The automated utilization data will provide information about all provider visits and hospitalizations at health plan facilities or paid for by the health plan. Visits for back pain can be identified by the diagnoses listed for every visit and hospitalization. Imaging studies of the lower back can also be identified by specific procedure codes.

Visits to non-study massage therapists and providers not covered by the health plan will be estimated from the interview data for the time period since the previous interview, i.e., the 10-week interview will request information about out-of-plan utilization since participants were randomized to study treatment, the 26-week interview will ask about the previous 16 weeks, etc. These data will permit determination of the percentage of participants in each treatment group who had any visits for low back pain during and following the intervention period, as well as calculation of the mean number of visits. Current use of medications of all types used for low back pain will be captured in the follow-up interviews.

Costs of back-related care will be estimated by assigning dollar values to each service. Costs of specific services (e.g., visits, imaging studies, medications) will be determined using data from Group Health's cost management information system which defines cost based on standard relative value units (RVUs) assigned to actual department RVUs produced. Costs of the massage interventions will be distinguished from the costs of all other back pain-related services to permit separate comparisons among the treatment groups of the costs of the interventions themselves, of the non-intervention back care costs incurred during the 10-week treatment period, and of the total back pain-related costs incurred over the 42 week period between completion of the study interventions and the end of the 52 week follow-up period.

### Data Collection and Management

We will collect information on the outcomes at every stage of our recruitment, randomization, and treatment process so we can report patient flow according to the CONSORT guidelines [22]. Specifically, we will record the number of invitation brochures sent, the number of responses received, the resolution of these responses (ineligible, refused, eligible and randomized, other), the number of participants assigned to a massage treatment who were actually treated, the number of visits made, the number of participants providing follow-up data by group at each follow-up, the number of participants completing the trial, and the number of withdrawals due to: ineffective treatment, adverse experiences, loss to follow-up, or other causes.

We will implement procedures to ensure that randomization is proceeding as planned, recruitment is on track, clinic data collection forms are accurately entered into databases, the computer assisted telephone interviewing (CATI) system is storing data correctly, and data can be accurately transferred and retrieved as needed. We will develop a relational database to track information on every stage of recruitment, randomization, treatment process, and outcomes assessment so we can report patient flow automatically and in an integrated fashion using standard, automated reports. To ensure accurate transfer of information, we will use electronic methods to routinely transfer data from the CATI database to the relational database. All data system processes will be thoroughly tested prior to the start of recruitment.

The CATI programs will contain range and logic checks. Appropriate information collected on the massage treatment visit forms will be entered into a computer database that also contains range and logic checks. Data will be examined for completeness using computer programs developed specifically for that purpose. In addition, we will test all analytic programs to ensure that the analyses are accurate. Procedures to protect the confidentiality and integrity of the databases and paper systems will be reviewed by the biostatistician prior to patient recruitment and periodically during the study.

To maintain the confidentiality of patient-related information in the database, unique participant study numbers will be used to identify patient outcomes and treatment data. The password security system will assign appropriate levels of computer privileges to different groups of database users. This will ensure that all masked personnel remain masked to treatment group.

Computer files with patient names will be password protected with access restricted to staff using this information to recruit patients, obtain follow-up data, and interact with any patients reporting adverse events. We will pre-test the procedure for sending the randomization information to the massage therapist and regularly audit treatment visit forms to ensure that participants being treated by massage therapists receive the treatment to which they were assigned and that the protocol is being correctly followed. During the treatment phase of the trial, we will continually monitor the massage therapists and provide focused booster training as needed. The massage therapists will mail copies of Visit Forms (identified only by study ID number and patient initials) to the research team after each participant has completed treatment. After being audited to ensure adherence to the protocol, charts will have initials removed, and again stored in locked filing cabinets accessible only to study personnel. Finally, all analysis data files will be password protected. Full data backup procedures will be performed nightly.

### Protection of human subjects and assessment of safety

#### Protection of human subjects

This study protocol was approved by the Institutional Review Board of Group Health Cooperative.

#### Data Safety Monitoring Body

Given the favorable safety profile from previous studies of massage coupled with the small numbers of adverse events reported in the literature, this trial will be monitored for safety by an independent Data Safety Monitoring Body (DSMB) comprised of a biostatistician, primary care physician and massage therapist. The DSMB's job will be to evaluate the adverse experience data we will provide them on a regular basis to protect the safety of the study participants. Based on the observed adverse effects of the treatments under study, the DSMB will make recommendations on a regular basis to the P.I. and the Office of Clinical and Regulatory Affairs at the National Center for Complementary and Alternative Medicine (NCCAM) regarding continuation, termination, or other modifications of the trial.

#### Adverse Events

Participants will be asked about adverse experiences at each massage visit and during the 10-week telephone interviews. We will define an adverse experience as any unfavorable and unintended sign, symptom or disease temporally associated with the use of the massage treatments that could reasonably be related to the treatments. Because massage has relatively short-term physiological effects, we will not report adverse events that first manifest more than one week after a participant's final treatment (or more than 10 weeks after randomization for the usual care control group). If a participant develops a Serious Adverse Experience (i.e., any adverse event occurring during treatment that results in any of the following outcomes: death, a life-threatening adverse event, inpatient hospitalization or prolongation of existing hospitalization, a persistent or significant disability/incapacity, or cancer) during the 10 weeks after randomization, it will be promptly (within 7 days) reported to the chair of the Data Safety Monitoring Body. If the Adverse Experience is, at least possibly, due to massage, it will also be promptly reported to the Group Health IRB and to NCCAM's Office of Clinical and Regulatory Affairs. In addition, we will summarize all adverse experiences in the trial in our routine reports to our DSMB and IRB.

#### Stopping rules

The trial will be stopped only if the Data Safety Monitoring Body (DSMB) believes there is an unacceptable risk of serious adverse events in one or more of the treatment arms. In this case, the DSMB could decide to terminate one of the arms of the trial or the entire trial.

We are not proposing to perform an interim analysis for efficacy and therefore have established no formal stopping rules. Even if the two massage treatments reduce pain and dysfunction (the primary outcomes of this trial) more than usual care, we do not consider this an important enough reason to end the trial early. Previous trials of massage have been plagued by small sample sizes and we think it is important that this study be large enough and long enough to clearly evaluate the effectiveness of massage.

### Statistical Issues

#### Sample size and the detectable difference

Because patient function (or dysfunction) is generally considered the more consequential of our two primary outcome measures, we want to ensure that our sample size estimates provide adequate power to detect a clinically significant difference of 2.0 points between groups on the Roland Disability scale [10]. To obtain outcomes data for 120 subjects per group, assuming a 10% loss to follow-up rate, we would need to randomize 133 participants. Our target sample size of 399 randomized individuals (133 per group) was computed using assumptions described below.

To protect against multiple comparisons, we will use Fisher's protected least significant difference approach which has been shown to have desirable properties when there are three groups [23]. This approach makes pair-wise comparisons between the three treatment groups only if the overall F-test is significant. The power of the overall analysis depends on how the means from the three treatment groups differ. We therefore assumed that the FSM group would be one Roland point superior to the RM group, which would, in turn, be one Roland point better than the UC group (giving a difference of 2 points between the FSM and UC groups).

Our estimates of the standard deviations of our primary outcome measures (adjusted for pre-randomization baseline values) derive from analyses of covariance of 8-week follow-up data from 92 study participants in our pilot study evaluating acupuncture for chronic low back pain: Roland SD = 4.65 and Bothersomeness SD = 2.71. Comparable standard deviation estimates at the end of the 10-week treatment period for 83 participants in our pilot study of CAM therapies for chronic low back pain, were 12%-15% lower. Thus, the standard deviation estimates we used for calculating statistical power in the proposed trial are probably conservative, likely underestimating actual power.

With 120 respondents per group (using two-sided significance level of 0.05) the omnibus F-test for the Roland scale will have 85% power for detecting a statistically significant difference among the three treatment means. If this omnibus test is statistically significant, as appears likely given our previous studies of FSM and those of others, we will address each Specific Aim of the study by comparing specified pairs of means, as discussed below. We will have 91% power to detect a pairwise difference of 2.0 Roland units.

For our other primary outcome measure, symptom bothersomeness, we want to be able to detect a minimal clinically important difference of 1.5 points on the 0 to 10 scale [10]. With data for 120 participants per group we will have over 99% power for detecting a minimal clinically significant difference among the three treatment means (assuming they are equally spaced and with 1.5 points between the extremes). In pairwise comparisons we will have 99% power to detect a 1.5-point difference on the Bothersomeness scale.

The power calculations are based on simple comparisons of the follow-up scores at a single point in time (ten weeks after randomization) with adjustment for baseline values using analysis of covariance. We also plan to adjust for other baseline characteristics (e.g., age, gender, and baseline covariates found predictive of 10-week outcomes). Inclusion of such baseline covariates can also improve precision of the variance estimate and therefore increase power.

#### Statistical Analysis

The primary statistical analyses will directly address Aim 1 (to determine if RM is an effective treatment for chronic low back pain) and Aim 2 (to determine if FSM is more effective than RM). A separate analysis will address the third aim, to explore the ability of psychosocial, and clinical baseline characteristics of patients to predict which of them will benefit most from each type of massage and which components of each treatment are associated with superior outcomes. These analyses are described below.

We will use an intent-to-treat approach for all analyses, i.e., individuals will be analyzed by the group to which they were randomized, regardless of participation in any treatment sessions. This minimizes biases that often occur when participants not receiving assigned treatments are excluded from analyses. We will use a standard linear regression mean model (ANCOVA) of the form:



where *Y*(*t*) is the response at follow-up time *t*, *Baseline *is the pre-randomization value of the outcome measure, *Trt *includes dummy variables for the full relaxation massage and focused structural massage groups, *Time *is a series of dummy variables indicating the follow-up times, and *z *is a vector of covariates representing other variables being adjusted for. (Note that α_1_, α_2_, α_3_, and α_4 _are vectors.) The referent group in this model is the usual care group at the first follow-up time. The models will be fitted using Generalized Estimating Equations (GEE) to take into account possible correlation within individuals over time [24]. GEE protects against misspecification of the correlation within a participant's scores over time. Such analyses can incorporate data for every time point at which a patient completes a follow-up interview. However, it is important to first assess whether there is differential dropout by either assigned treatment or symptom or function level. If differential dropout occurs, we will use the method of Little to rectify possible biases that could occur [25].

Both primary outcomes (function and symptoms) will be tested at the 0.05-level, because they address separate scientific questions. Analyses of both outcomes will be reported, imposing a more stringent requirement than simply reporting a sole significant outcome. Arguments against adjusting for multiple comparisons in this situation have been made by Rothman) and others [26-28].

If we find a significant overall difference, we will localize the difference among the three groups using pairwise comparisons in accordance with our specific aims. For Aim 1, we will compare the RM group with the UC group. For Aim 2, we will compare the RM group with the FSM group. We will also compare the FSM group and the UC group, although this is not included as a specific aim.

We will consider interactions of treatment and the baseline value that would indicate the effect of treatment depends on status at baseline. We will also test for significant interactions of treatment with other variables (e.g., age, race, gender, and pain below the knee but not meeting criteria for sciatica) to determine if treatment differences are modified by these variables. To explore whether baseline characteristics predict which treatment performs better (Aim 3), we will examine our main outcome measures separately by subgroups using both tabular and graphical methods. We will begin with simple exploratory data analysis approaches, because we have no initial hypotheses about which characteristics will predict treatment success. Exploratory analysis will examine the univariate relationships between potential baseline predictor variables and outcomes of the treatments. We may also use more sophisticated statistical methods (e.g., latent class analysis) to identify individuals with different patterns of baseline characteristics that respond differently to the treatments.

We will also analyze secondary outcomes for Aims 1 and 2, including costs of back care, general health status (SF-36), depression, perceived stress, fear avoidance, disability days, and satisfaction with care. An analysis of costs may entail a transformation (e.g., log cost) but can be modeled using linear regression without a baseline value for costs since they will be unknown. We will adjust for age and other factors that may affect costs. We recognize that we will have low statistical power to detect even moderate cost differences among the groups. For binary outcomes (e.g., medication use) we will use logistic regression. Despite the multiplicity of analyses, we will focus our main analyses on treatment effects on each of the five outcome domains recommended for studies of back pain [8].

We are interested in the effect of individual massage therapists on the outcomes. Because each massage therapist will deliver both RM and FSM treatments, we can compare treatment effects within each massage therapist. While differences would have to be extremely large to detect a significant difference for a particular therapist, we may be able to determine whether there is significant variation across massage therapists that cannot be explained by random variation.

Finally, we will conduct exploratory analyses using the treatment log data to examine relationships among underlying conditions, treatments used and treatment outcomes. For the FSM treatment group, we will describe the frequency with which each specific technique was used and the relationship between the underlying condition and the technique(s) selected, using chi-square tests. We will then examine the relationship between the underlying condition and the likelihood of a good outcome and whether this relationship is affected by the specific treatment(s) employed. For these analyses we will use standard multivariate techniques (e.g., analysis of covariance, logistic regression, GEE). For the RM group, which follows a relatively fixed treatment protocol, we will only examine the relationship between the underlying condition and treatment outcomes.

## Competing interests

The authors declare that they have no competing interests.

## Authors' contributions

DC, KS, JK, RD participated in the conception and design of the trial, in plans for the analysis of the data, and in drafting and/or critically reviewing the manuscript. JE participated in the conception and design of the trial and in critically reviewing the manuscript. SH and AC participated in the design of the trial and in critically reviewing the manuscript. All authors read and approved the final manuscript.
